# Functions of p120-catenin in physiology and diseases

**DOI:** 10.3389/fmolb.2024.1486576

**Published:** 2024-10-21

**Authors:** Xin Jin, Ting Lin, Yunjuan Wang, Xiaoqian Li, Yanhong Yang

**Affiliations:** ^1^ The First Affiliated Hospital (The First School of Clinical Medicine), Guangdong Pharmaceutical University, Guangzhou, China; ^2^ Guangdong Metabolic Diseases Research Center of Integrated Chinese and Western Medicine, Guangdong Pharmaceutical University, Guangzhou, China

**Keywords:** P120, cell-cell adhesion, cancer, inflammation, non-coding RNAs

## Abstract

p120-catenin (p120) plays a vital role in regulating cell-cell adhesion at adherens junctions, interacting with the juxtamembrane domain (JMD) core region of E-cadherin and regulates the stability of cadherin at the cell surface. Previous studies have shown significant functions of p120 in cell-cell adhesion, tumor progression and inflammation. In this review, we will discuss recent progress of p120 in physiological processes and diseases, and focus on the functions of p120 in the regulation of cancer and inflammation.

## 1 Introduction

p120-catenin was initially considered as a tyrosine kinase substrate and was later shown to interact with E-cadherin (Reynolds et al., 1994). The binding of p120 to JMD in the cytoplasmic tail of cadherin promotes the formation of dimers on the plasma membrane of E-cadherin and maintains the surface stability of the cadherin-catenin cell-cell adhesion complexes (Ishiyama et al., 2010; Vu et al., 2021; Su et al., 2024). Defects in the p120 binding site or the absence of the p120 serine/threonine phosphorylation would make the cell-cell adhesion unstable (Fukumoto et al., 2008). In addition to its crucial role in increasing the stability of cadherin, p120 also reduces the sensitivity of the cadherin-catenin complex to endocytosis, ubiquitination, and proteosome destruction (Ireton et al., 2002; Davis et al., 2003; Miyashita and Ozawa, 2007). Loss of p120 affects cell-cell adhesion, contributing to the dysfunction of adhesion junction (Smalley-Freed et al., 2010; Schackmann et al., 2013). p120 has other physiological functions such as maintaining epidermal physiological function (Xie et al., 2018), regulating signal transduction (Xiao et al., 2007; Stepniak et al., 2009), participating in embryonic development (Oas et al., 2010; Pieters et al., 2016; Hernández-Martínez et al., 2019) and alleviating pulmonary fibrosis (Zhang et al., 2019). Non-coding RNAs play an important role in the regulation of p120 and are involved in physiological and disease processes (Xiong et al., 2024; Deng et al., 2024; Wu et al., 2016).

p120 can inhibit tumor growth and invasion and play a crucial role in tumor progression as a tumor suppressor (Stairs et al., 2011). Downregulation of p120 is a common feature of cancer and causes epithelial cells to lose polarity and increase the proliferation, survival, and invasion of epithelial cells (Venhuizen et al., 2020; Rajeev et al., 2024). Paradoxically, in addition to this inhibitory effect on tumors, p120 also has a tumor-promoting effect, promoting tumor migration and invasion (Tenhagen et al., 2016; Bartolomé et al., 2024; Wang et al., 2024). Glutathionylation and phosphorylation of p120 can increase the migration and invasion of cancer cells (Kaszak et al., 2020; Kukulage et al., 2023). p120 is also essential for inflammation responses and immunity of the organisms. It has been reported that the nuclear factor-kappa B (NF-κB) signaling was activated in the p120-conditioned knockout mice, leading to immune infiltration and expression of pro-inflammatory cytokines (Perez-Moreno et al., 2006; Perez-Moreno et al., 2008). As a key regulator of the NLRP3 inflammasome, p120 is also involved in the anti-inflammatory response by protecting the structure and function of mitochondria to regulate NLRP3 assembly and activation (Liu et al., 2019; Kanmani et al., 2023). p120 positively regulates the production of type-I interferon (IFN-I), which is critical for the host to eliminate the invading viruses (Wu et al., 2023). Knocking out of p120 in mice leads to more infiltration of immune cells and a cascade of inflammatory responses (Wu et al., 2023; Chignalia et al., 2015).

A growing body of research sheds light on the function of p120 in physiology and disease, and we will review the recent findings and progresses of the underlying mechanisms.

## 2 Functions of p120-catenin in physiological processes

It has been demonstrated that the JMD of cadherin is required for recruiting p120 to the adherens junctions (Daniel and Reynolds, 1995; Yap et al., 1998; Thoreson et al., 2000), and the expression of different cadherin cytoplasmic domains and mutation analysis of the JMD has demonstrated that the p120-catenin-E-cadherin interaction is necessary for increased cell adhesions (Ferber et al., 2002; Hartsock and Nelson, 2008). Several studies have indicated that loss of p120 could destabilize adherens junctions and affect intercellular adhesion. The function of p120 in the intestine is vital to epithelial homeostasis and survival, and intestinal-specific p120 ablation in mice could cause cell-cell adhesion defects and reduce the levels of E-cadherin and β-catenin, leading to epithelial barrier defects (Smalley-Freed et al., 2010). In addition, p120 ablation in the skin epidermis of mice contributes to decreased levels of cadherins and catenins at sites of intercellular contacts (Perez-Moreno et al., 2006). *Leptospira* interrogans could disrupt epithelial adherens junctions and spread *in vivo* by inducing p120 degradation (Tokumon et al., 2023).

Microtubules are known to interact with the cell-cell adhesion machinery, and p120 in the cytoplasm can be directly or indirectly linked to the microtubule network (Kourtidis et al., 2013). In addition, p120 facilitates the stabilization, bundling, and tethering of microtubules to the connection points of the cell-cell adhesions through a binding partner called PLEKHA7 (Franz and Ridley, 2004; Yanagisawa et al., 2004; Ichii and Takeichi, 2007; Meng et al., 2008; Pulimeno et al., 2010). Studies have shown that phosphorylation of p120 participate in cell-cell adhesion, and the serine/threonine phosphorylation of p120 at the plasma membrane could influence the dynamics of E-cadherin in cell-cell junctions, playing a critical role in regulating cadherin activation and adhesion strengthening (Ozawa and Ohkubo, 2001; Fukumoto et al., 2008; Petrova et al., 2012). The Rho GTPases RhoA, Rac1 and Cdc42 can modulate cadherin-mediated adhesion and cell-cell junction formation by manipulating the actin cytoskeleton, and p120 may modulate the activity of RhoA to regulate cell adhesion, which is thought to be required for an earlier step in the junction formation (Anastasiadis, 2007; Gama and Schmitt, 2012; Menke and Giehl, 2012).

In addition, p120 has other vital physiological functions. Mouse tissue-specific deletion of p120 suggests that p120 is critical for the normal morphogenesis, and it has been reported that p120 is necessary to mammary morphogenesis and terminal end bud function in mice, and that reduction of p120 in the developing mammary gland defers ductal outgrowth (Kurley et al., 2012). Conditional p120 ablation in mice affects the acinar differentiation of the salivary glands, and there are obvious defects in cell-cell adhesion and epithelial morphology in these mice (Davis and Reynolds, 2006). p120 is essential for early tubular and glomerular morphogenesis, and loss of p120 in the renal mesenchyme contributes to hypoplastic kidneys and abnormal tubular structural morphology. (Marciano et al., 2011). Conditional knockdown of p120 in the mouse epidermis not only leads to increased epidermal proliferation, but also leads to decreased epidermal differentiation and impaired permeability barrier function, suggesting that p120 is necessary to inhibit epidermal proliferation, promote epidermal differentiation and maintain permeability barrier function of the epidermis (Xie et al., 2018). p120 is also required for regulating mammalian vascular development, and mice lacking endothelial p120 could result in a decrease in endothelial cadherins and defects in cell proliferation, affect microvascular morphogenesis and vascular integrity, and lead to embryonic lethality beginning around E12.5 (Oas et al., 2010). Moreover, p120 can regulate the germ layer differentiation, and the absence of p120 in mouse embryos can lead to embryonic arrest at midgestation (Hernández-Martínez et al., 2019). Non-coding RNAs can affect the expression of adhesion molecules in a post-transcriptional manner, playing a crucial role in endothelial function and vascular barrier integrity (Maucher et al., 2021). A new study have shown that miR-194-3p directly targets p120-catenin and regulates its expression, thereby altering the expression of β-catenin, which has a key impact on the epithelial-mesenchymal transition process in the embryonic epicardial cells through the cell adhesion mechanism (Xiong et al., 2024).

Furthermore, p120 binds to E-cadherin to regulate the differentiation of mouse stem cells into the primitive endoderm and maintain endodermal cell polarity (Pieters et al., 2016). In bleomycin (BLM)-induced pulmonary fibrosis mouse, p120 plays a vital role in regulating the process of pulmonary fibrosis via the SMAD and NF-κB pathways, and the absence of p120 can alleviate pulmonary fibrosis and lung fibroblast differentiation in mouse (Zhang et al., 2019).

## 3 p120-catenin and diseases

### 3.1 p120-catenin in cancer

Normal cells inhibit growth and migration by adhering to each other, and these properties are gradually lost in tumor cells, resulting in increased cell proliferation and mobility (Kourtidis et al., 2013). E-cadherin plays an essential role in homeostasis and normal development, it is also a tumor suppressor, absence of human E-cadherin is thought to be related to poor prognosis in a variety of cancers (Dai et al., 2017; Xie et al., 2017; Yazdani et al., 2018). p120 is required for cadherin stability, which can stabilize E-cadherin on the plasma membrane by suppressing endocytosis and proteasome disruption (Ireton et al., 2002; Davis et al., 2003; Miyashita and Ozawa, 2007). E-cadherin dimerization on the plasma membrane requires p120-catenin binding to the JMD, and knockdown of p120 results in the absence of junctional E-cadherin (Vu et al., 2021). A recent study suggests that p120 is extremely susceptible to glutathionylation at C692, resulting in its separation from E-cadherin and distribution to the cytoplasm, followed by E-cadherin ubiquitination and degradation in the proteasome, thereby increasing cell migration and invasion under oxidative stress (Kukulage et al., 2023). Because of its ability to modulate E-cadherin function, p120 can exert an inhibitory effect on tumor progression by inhibiting proliferation and invasion (Stairs et al., 2011).

As a tumor marker, p120 is lower expressed in many cancers, such as tumors of the colon, stomach, breast, lung and pancreas (Bremnes et al., 2002; Thoreson and Reynolds, 2002; Wang et al., 2006). Moreover, p120 is a clinically useful diagnostic biomarker, such as for the diagnosis of breast lobular tumors and milk ductal tumors, and the differentiation of plasmacytoid and sarcomatoid variants from conventional urothelial carcinoma (Dabbs et al., 2007; Li et al., 2014; Acosta et al., 2021). Among the 47 clinical cases of oral cancer, 36% showed low expression of E-cadherin, 34% showed low expression of p120, and 80.8% of cases found abnormal cytoplasmic localization of p120 (Salam et al., 2023). Loss of p120 also correlates with cancer poor prognosis, as tumors with decreased expression of p120 have a higher chance of metastasizing and poor survival rate. The altered membrane expression level of p120 acted as an independent prognostic factor for esophageal squamous cell carcinoma patient survival and for the migration and invasive behavior of the disease (Chen et al., 2015). Reduced expression of p120 is closely related with the survival of patients and the aberrant expression of p120 is an independent prognostic factor for intrahepatic cholangiocarcinoma (Zhai et al., 2008). Absence of p120 expression with decreased survival in bladder cancer (Shimazui et al., 1996), and altered p120 expression may be associated with poor prognosis of colorectal cancer (Gold et al., 1998). Reduction or absence of p120 in primary invasive ductal breast cancer is significantly associated with a worse prognosis (Kurley et al., 2020).

**FIGURE 1:**
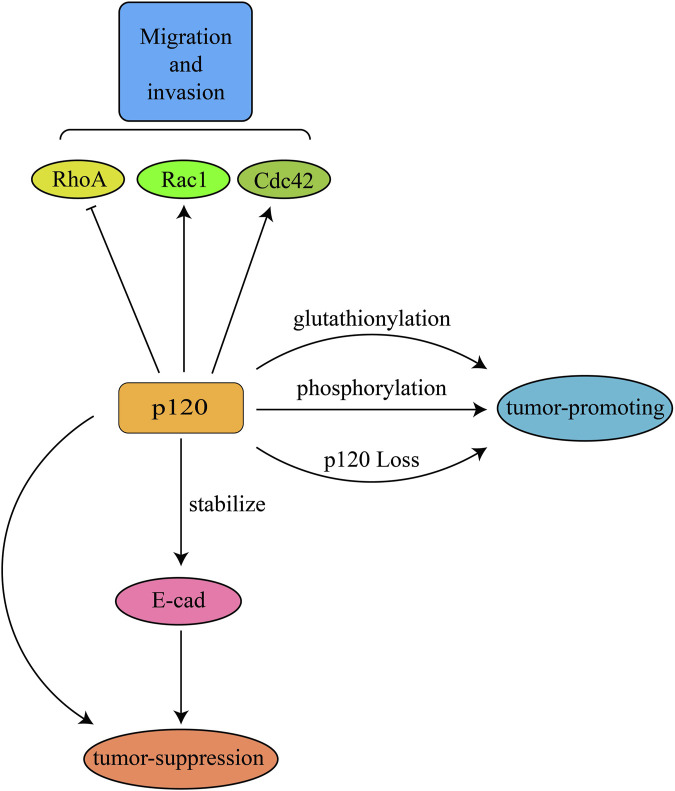
The dual function of p120 in tumor suppression and promotion.

Several studies have demonstrated that absence of p120 contributes to pro-tumorigenesis events. In a conditional mouse model of noninvasive breast cancer, deletion of p120 could lead to the formation of stromal-dense tumors and metastasize to the lungs and lymph nodes (Schackmann et al., 2013). In tamoxifen-inducible mouse model, limited p120 knockout in the intestine could contribute to the formation of adenoma through an indirect effect caused by p120 deletion rather than cellular autonomy (Smalley-Freed et al., 2011). The deletion of p120 in the salivary glands of mice showed that acinar differentiation was entirely blocked, leading to a gland composed fully of ducts and severe defects in adhesion, cell polarity and epithelial morphology, which was similar to high-grade intraepithelial neoplasia (Davis and Reynolds, 2006). Knockout of p120 in oral squamous cell carcinoma stimulated epidermal growth factor-induced nuclear phospholipase C-γ1 signaling, resulting in cell proliferation and poor cell differentiation *in vivo* (Li et al., 2020). Conditional deletion of p120 have displayed pretumor and tumor lesions in the oral cavity, esophagus and squamous forestomach of mice, leading to invasive squamous cell cancer (Stairs et al., 2011). In a study of p120 expression in oral squamous cell carcinoma and apparently normal mucosa adjacent to oral squamous cell carcinoma, p120 expression in oral squamous cell carcinoma was reduced and mislocalized, shifting from membrane to cytoplasmic expression (Rajeev et al., 2024). Furthermore, p120 downregulation may lead to tumor progression and metastasis by reducing β-catenin and E-cadherin expression, and altering the activities of Cdc42, Rac1 and RhoA (Liu et al., 2009). The above results demonstrate that p120 can exert tumor suppressive function, and the absence of p120 results in the development of neoplastic lesions (Figure 1).

Surprisingly, a range of evidence suggests that p120 also has tumor-promoting effects and crucial role in the migration and invasion of tumors. The expression of p120 isoform 1 is regulated by desmocollin-1, and downregulation of desmocollin-1 leads to increased expression of p120 isoform 1 in epithelial cells and alteration of cellular location, thereby increasing cell migration and invasion (Bartolomé et al., 2024). By regulating intracellular calcium ion levels and participating in microtubule formation, p120 can promote the process of glioma cell invasion and proliferation (Wang et al., 2024). It has been reported that p120 increases the migration and invasion ability of the epidermal growth factor receptor 2-induced breast cancer cells through inducing Rac1 and Cdc42 activity (Johnson et al., 2010). p120 can facilitate the transformed growth of human breast cancer cells through Rac1 activation and Rac1-mediated MAPK signaling, and modulate the invasion and migration of GnRH-induced human ovarian cancer cells through modulating Rac1 and Cdc42 (Soto et al., 2008; Cheung et al., 2010). It has also been reported that p120 binds to mesenchymal cadherins to activate Rac1 and increase the motility and invasiveness of the E-cadherin–deficient breast cancer cells (Yanagisawa and Anastasiadis, 2006). In addition, p120 is essential for anchorage-independent growth of tumor cells via regulating ROCK pathway, ablation of p120 completely blocked Rac1– and Src–mediated anchorage-independent growth (Dohn et al., 2009). In conclusion, p120 can bind to Rho to exert the effect of RhoGDI to inhibit Rho or activate Rac and cdc42 to promote tumor invasion and migration.

A number of studies have demonstrated that p120 can be phosphorylated in multiple serine, threonine and tyrosine residues, thereby affecting activation of cadherin (Mariner et al., 2001; Xia et al., 2003; Castaño et al., 2007; Fukumoto et al., 2008). More importantly, phosphorylation of p120 plays an important role in mediating cell adhesion, cell metastasis, and cell proliferation (Mendonsa et al., 2020). The level of phosphorylation of p120 was positively correlated with tumor aggressiveness in glioblastoma multiforme (Huveldt et al., 2013). Studies have demonstrated that tyrosine and threonine phosphorylation of p120 is elevated in renal and breast tumor tissue samples, and tyrosine phosphorylation is necessary for its pro-tumorigenic potential (Kourtidis et al., 2015). Overexpression of receptor-type tyrosine-protein phosphatase zeta regulated the phosphorylation of p120/β-catenin to enhance oral submucous fibrosis malignancy (Ma et al., 2022). In potentially malignant oral lesions, high levels of phosphorylated p120 expression on cell membranes increase the incidence of oral squamous cell carcinoma and promote invasion (Ma et al., 2012). In addition, p120-catenin isoform 3 regulates the nuclear export of Kaiso and increases invasion in lung cancer cells via a phosphorylation-dependent mechanism, and the phosphorylation of serine and threonine in p120 could augment the invasion ability of the lung cancer cells (Zhang et al., 2011).

**FIGURE 2:**
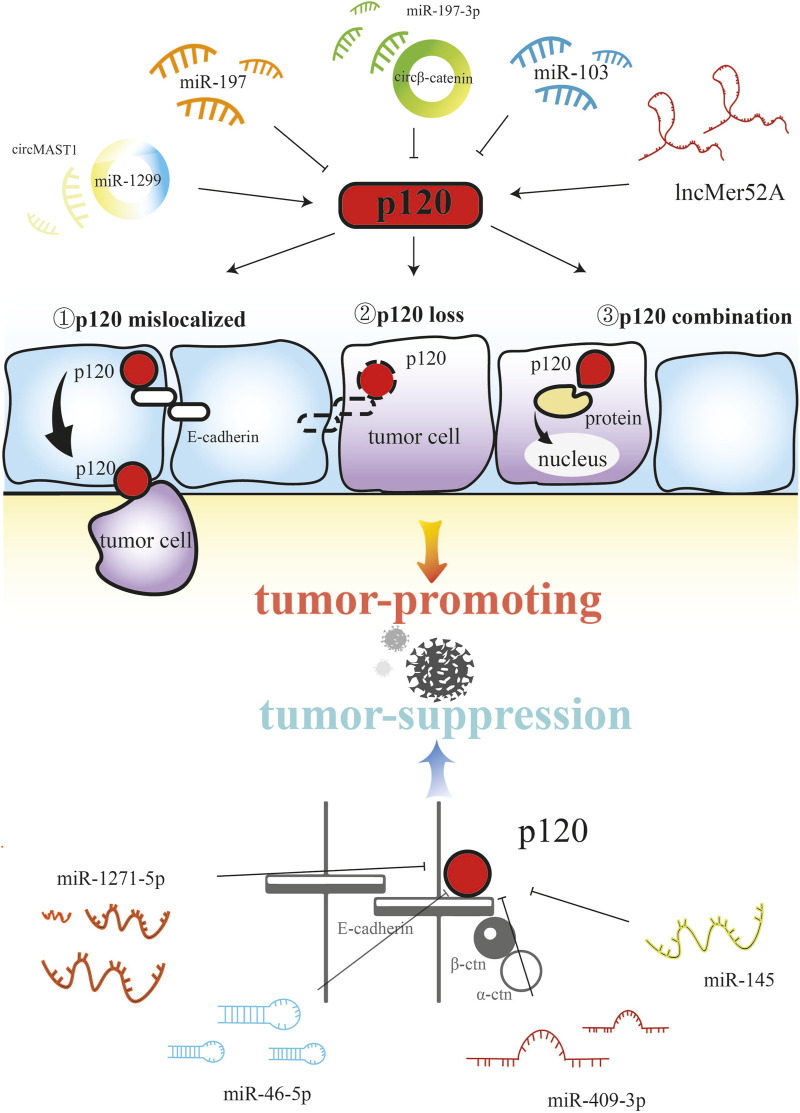
Non-coding RNAs affect tumorigenesis through the regulation of p120.

Non-coding RNAs play a comprehensive regulatory role in various biological processes (Omran et al., 2024; Mohamed et al., 2022; Nemeth et al., 2024), and by targeting p120, non-coding RNAs play different roles in tumorigenesis and development (Figure 2). circβ-catenin is a novel oncogene in colorectal cancer that can be used as a poor prognostic marker in colorectal cancer patients. circβ-catenin directly binds to miR-197-3p, and then inhibits the target p120, which ultimately promotes the proliferation and metastasis of colorectal cancer (Deng et al., 2024). miR-223 directly targets p120 to downregulate the expression of p120 thereby reducing cell-cell adhesion, enhancing RhoA activity, and activating β-catenin signaling to promote colon cancer cell invasion and metastasis (Liu et al., 2017). miR-103 secreted by hepatocellular carcinoma cells can increase vascular permeability and inhibit the expression of p120 in vascular endothelial cells, thereby promoting tumor metastasis (Fang et al., 2018). circMAST1 promotes the expression of p120 by directly binding to miR-1299, thereby maintaining hepatocellular carcinoma invasion and proliferation (Yu H. et al., 2020). miR-197 can target and reduce the endogenous p120 expression to promote the migration and invasion of pancreatic cancer cells (Hamada et al., 2013). lncMER52A can promote the progression of hepatocellular carcinoma cells by blocking p120 ubiquitination–proteasome degradation and stabilizing p120 to activate Rac1 and Cdc42 (Wu et al., 2020). In addition, miR-409-3p directly targets and inhibits the expression of p120 to suppress the metastasis and invasion of osteosarcoma (Wu et al., 2016). miR-145 inhibits cytoplasmic expression of p120 and rescues membrane localization of E-cadherin and p120 by downregulating N-cadherin, thereby inhibiting the invasion of gastric cancer cells (Xing et al., 2015). miR-1271-5p exerts anti-cancer effects by directly targeting and downregulating p120 in regulating the proliferation, migration, apoptosis and invasion of endometrial carcinoma (Wei et al., 2021). miR-96-5p inhibits p120 expression and p120-mediated Wnt/β-catenin signaling after transcription, thereby inhibiting *in vitro* metastasis of breast cancer cells (Gao et al., 2020). Overall, non-coding RNAs play a promoting and inhibitory role in tumorigenesis and development, mainly through targeted regulation to reduce the expression of p120.

### 3.2 p120-catenin in inflammation and immunity

Inflammation is the basis of various physiological and pathological processes. An increasing number of research has manifested that p120 plays a critical role in the inflammatory response of many tissues and organs. NF-κB is an essential transcription factor associated with inflammation and various diseases, and various stimuli can activate NF-κB and produce transcriptional activity, which modulate the expression of proinflammatory genes including chemokines, cytokines, and adhesion molecules (Barnabei et al., 2021; Yu X et al., 2020; Lawrence, 2009).

There has been evidence that NF-κB activity is increased in mice with p120 skin-specific deficiency, and loss of p120 also increased the number of epidermal inflammatory cells by activating NF-κB signaling, thereby inducing pro-inflammatory cytokine production (Perez-Moreno et al., 2006; Perez-Moreno et al., 2008). In addition, p120 could prevent the production of intermittent cyclic mechanical tension-induced inflammatory mediators and attenuates the inflammatory responses of the human bronchial epithelial cells by inhibiting the NF-κB signaling pathway (Zhang et al., 2016; Xu et al., 2017). p120 plays a regulatory role in E-cadherin in chronic rhinosinusitis, thereby attenuating the disruption of nasal mucosal epithelial barrier by inflammatory mediators (Li et al., 2022). Furthermore, p120 could protect human brain microvascular endothelial cells (HBMECs) and improve blood-brain barrier dysfunction by inactivating NF-κB to inhibit the LPS-induced inflammatory response (Liu et al., 2015).

NLRP3 inflammasome is closely related to the regulation of inflammation and antiviral responses (Zhao and Zhao, 2020), while mitochondrial reactive oxygen species (ROS) can lead to the activation of NLRP3 inflammasome (Dan Dunn et al., 2015). Recent findings have displayed that p120 plays a critical role in the regulation of the NLRP3 inflammasome in inflammatory response and polymicrobial sepsis (Kanmani et al., 2023). p120 is involved in the inhibition of NLRP3 inflammasome activation and can suppress the release of IL-1β and IL-18 and the expression of active Caspase-1 by blocking mitochondrial ROS generation, whereas absence of p120 could enhance the activation of NLRP3 inflammasome in macrophages and significantly increase the secretion of IL-18 and IL-1β in mouse lungs (Kanmani et al., 2023). Another study showed that the depletion of p120 in murine alveolar epithelial cells during mechanical stretching significantly increased the expression of caspase-1 and the activation of NLRP3, aggravating mitochondrial dysfunction, and the results suggest that p120 prohibited the activation of NLRP3 to protect the structure and function of mitochondria by suppressing TLR4 pathway and ROS production in ventilator-induced lung injury in mice (Liu et al., 2019).

Studies have found that p120 has an important positive modulatory role in innate antiviral immunity. It has been reported that the production of IFN-I is vital for the host to eliminate the invading viruses, and the ectopic expression of p120 enhances the production of IFN-I, greatly reducing viral replication; mice knock out of p120 are more susceptible to vesicular stomatitis virus infection, less IFN-I production and greater infiltration of immune cells (Wu et al., 2023). Mechanistically, p120 stabilizes the Serine/threonine-protein kinase 1-interferon regulatory factor 3 complex and increases interferon regulatory factor 3 activation to promote host antiviral responses (Wu et al., 2023). p120 also plays an important role in regulating innate immune function in the lungs. Mice with knockout of p120 in alveolar epithelial type II cells exhibit pulmonary epithelial barrier defects and severe lung inflammation manifested by an marked infiltration of inflammatory cells and activation of NF-κB, as well as increased expression of macrophage inflammatory protein-2, intercellular adhesion molecule 1 (ICAM-1) and TLR4 (Chignalia et al., 2015). Mouse lung endothelial cell p120 can reduce the inflammatory response of the lungs to endotoxins by inhibiting TLR4 signaling and regulate the innate immune function of the lungs, knockdown of p120 increased the expression of ICAM-1, neutrophil recruitment and production of pro-inflammatory cytokine (Wang et al., 2011). In addition, p120 is expressed in epithelial cells of diseased glomeruli and, together with β-catenin, is involved in the pathogenesis of cellular crescents or microadhesions in lupus-associated glomerulonephritis (Nakopoulou et al., 2002; Usui et al., 2003). Through the mechanism of signaling kinases of intercellular adhesion molecules, phosphorylation of p120 can affect IgG autoantibody production in patients with the immunoblistering skin disease pemphigus vulgaris to induce keratolysis dyshesion (Chernyavsky et al., 2008). p120 is highly expressed in the seminiferous epithelial cells of rats, and the loss of spermatogenic cells observed in orchitis rats is associated with early alterations in the expression and function of adherens junction molecules (Pérez et al., 2011). These findings suggest the essential regulatory role of p120 in innate immunity.

Deficiency of p120 was closely associated with increased pro-inflammatory activity in multiple tissues. The experimentally-induced loss of p120 expression increased pro-inflammatory adhesion molecules such as ICAM-1, VCAM-1, E-selectin and P-selectin at the transcriptional level, thereby promoting pro-inflammatory activity in human pulmonary artery endothelial cells (O’Donnell et al., 2011). As previously described, p120 was vital for epithelial homeostasis and survival, and absence of p120 in mouse intestinal epithelial cells could cause mucosal damage and inflammation, resulting in massive intestinal bleeding and death within the first 3 weeks of life (Smalley-Freed et al., 2010). Decreased expression of p120 at the edge of the ulcer mucosa has been reported in 100% of cases of active ulcerative colitis and 75% of cases of active Crohn’s disease (Karayiannakis et al., 1998).

## 4 The relationship between p120-catenin and inflammatory signaling pathways

### 4.1 Functions of p120-catenin in TLR4 signaling pathway

Toll-like receptor 4 (TLR4) is a member of the Toll-like receptors (TLRs) family that recognizes lipopolysaccharides (LPS) or bacterial endotoxins to mediate inflammatory responses and participate in innate immunity. Endogenous molecules or exogenous substances could activate TLR4 to induce a cascade of immune and inflammatory responses that were vital to innate immune responses against bacterial and viral infections (Kuzmich et al., 2017; Zhang et al., 2022). MyD88 is one of the Toll/IL-1 receptor (TIR) domain-containing adaptors, which regulates the TLR signaling pathway and is essential for the induction of inflammatory cytokines triggered by all TLRs (Takeda and Akira, 2004). TLR4 could increase the induction of pro-inflammatory cytokines through the MyD88-dependent pathway and could also use TIR domain-containing adaptor to induce TRIF but not MyD88 to enhance IRF3-induced IFN-I expression in the endosome (Duan et al., 2022).

Studies have demonstrated that p120 selectively modulates LPS-induced TLR4 signaling in macrophages, promotes TLR4 internalization under LPS stimulation, thereby inhibiting MyD88-mediated TLR4 signaling and the release of pro-inflammatory cytokines (Yang et al., 2014). In addition, p120 upregulates TRIF-mediated TLR4 signaling and IFN-β production by enhancing the endocytosis of TLR4 (Yang et al., 2014). Similar studies have shown that overexpression of p120 in lung endothelia of mice inhibits the TLR4-MyD88 interaction, while loss of p120 enhances LPS-induced binding between TLR4 and MyD88, which in turn regulates TLR4 signaling through the MyD88-dependent pathway, contributing to NF-κB activation and pro-inflammatory cytokine production; furthermore, p120 could prevent LPS-induced IL-1R–associated kinase-4 activation in mouse lung endothelial cells, which has been shown to enhance TLR4 signaling (Wang et al., 2011). These findings manifest that p120 plays an essential role in modulating TLR4 signaling by the MyD88-dependent pathway.

### 4.2 Functions of p120-catenin in RhoA/ROCK signaling pathway

Various signal transduction pathways in all eukaryotic cells are regulated by Rho GTPases, which play an important role in regulating actin cytoskeleton, microtubule dynamics, cell polarity, membrane transport pathways and transcription factor activity, and RhoA is the most well researched member of Rho GTPases (Etienne-Manneville and Hall, 2002). Rho kinase (ROCK) is a serine/threonine kinase that is an essential downstream effector of RhoA, and the RhoA/Rho-kinase pathway plays a crucial role in many cellular functions (Shimokawa et al., 2016).

Several studies have shown that RhoA/ROCK is also involved in the regulation of inflammatory responses. In human bronchial epithelial cell airway inflammation, p120-mediated NF-κB signaling activation was dependent on RhoA/ROCK axis, and exposure to cigarette smoke extract resulted in downregulation of p120 expression which in turn activates the NF-κB signaling pathway with increased levels of pro-inflammatory cytokines (Zhang et al., 2016). p120 also activates the NF-κB signaling pathway partially via RhoA in LPS-treated human bronchial epithelial cells (Qin et al., 2014). In addition, it has been shown that p120 was markedly reduced in human bronchial epithelial cells after scratching, while the reduction of p120 leads to an increase in IL-8 induced by the activation of NF-κB and IκBα phosphorylation; furthermore, the anti-inflammatory effects of p120 in human bronchial epithelial cells rely on the RhoA/ROCK axis to regulate NF-κB (Qin et al., 2015). p120-deficient epidermal cells could activate transcription factor NF-κB, triggering a series of pro-inflammatory NF-κB targets partly by regulating RhoA (Perez-Moreno et al., 2006). In summary, p120 and RhoA/ROCK have been displayed to be associated with inflammation, and p120 can participate in modulating the inflammatory response by regulating the RhoA/ROCK pathway to inhibit NF-κB signaling.

## 5 Conclusion and perspectives

**FIGURE 3:**
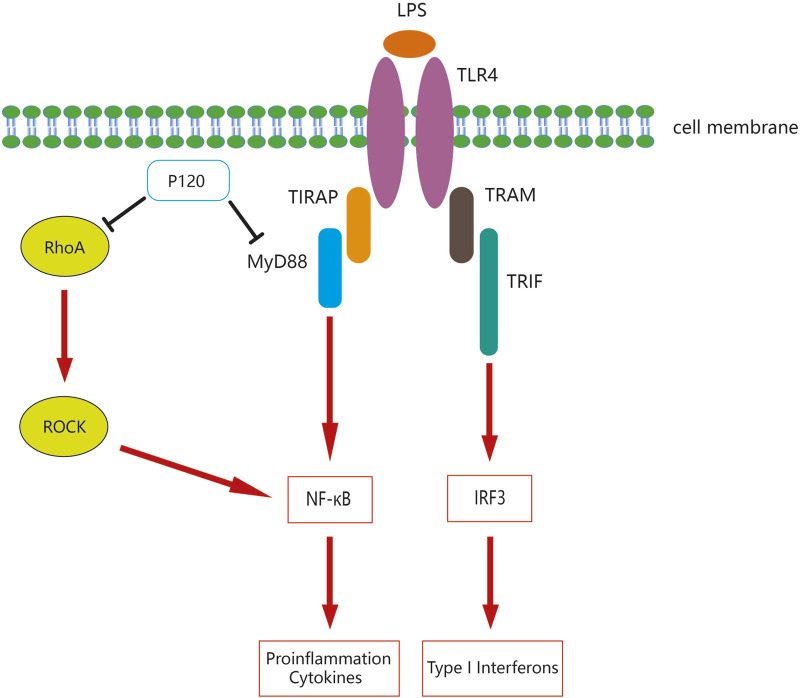
Mechanisms of p120 mediated regulation of the TLR4 signaling pathway and the RhoA/ROCK signaling pathway.

An increasing number of research has expanded the understanding of the function of p120-catenin, which is involved in a plethora of physiological processes. p120 participates in epidermal proliferation, epidermal differentiation, embryonic development and signal transduction. p120 is indispensable for the regulation of the occurrence and development of tumors, which can not only inhibit tumor progression, but also promote tumor development. The decrease in p120 expression is strongly associated with tumor prognosis, aggressiveness, and metastasis. By altering the activity of Cdc42, Rac1, and RhoA, and stabilizing E-cadherin, p120 plays a different role in tumor progression. As an important modulator of inflammation and immunity, p120 exerts anti-inflammatory and antiviral effects through different pathways. p120 can regulate the inflammatory response by modulating TLR4 signaling pathway and RhoA/ROCK signaling pathway (Figure 3). Moreover, p120 is also an important endogenous anti-inflammatory mediator. Many studies of conditioned knockout mice have shown that p120 deficiency is strongly associated with increased pro-inflammatory activity in a variety of tissues, manifested by the production of pro-inflammatory cytokine and increased inflammatory cell infiltration. The data from clinical trials is crucial to the study of p120, which allows us to better understand the physiological function of p120 and the pathogenesis of the disease. Although there are no clear reports on the interaction between tumor and immunity, it is believed that with the development of CRISPR-Cas9, single-cell sequencing, spatial transcriptomics and other technologies, p120 research can make breakthroughs in tumor-immune interactions, and the mechanism and function of p120 gene in physiology and disease can be detected faster and more accurately in the future.
